# Sensomics-Assisted Aroma Decoding of Pea Protein Isolates (*Pisum sativum* L.)

**DOI:** 10.3390/foods11030412

**Published:** 2022-01-30

**Authors:** Florian Utz, Andrea Spaccasassi, Johanna Kreissl, Timo D. Stark, Caren Tanger, Ulrich Kulozik, Thomas Hofmann, Corinna Dawid

**Affiliations:** 1 Chair of Food Chemistry and Molecular Sensory Science, TUM School of Life Sciences, Technical University of Munich, Lise-Meitner-Straße 34, 85354 Freising, Germany; florian.utz@tum.de (F.U.); andrea.spaccasassi@tum.de (A.S.); timo.stark@tum.de (T.D.S.); thomas.hofmann@tum.de (T.H.); 2 Leibniz-Institute for Food Systems Biology at the Technical University of Munich, Lise-Meitner-Straße 34, 85354 Freising, Germany; j.kreissl.leibniz-lsb@tum.de; 3 Chair of Food and Bioprocess Engineering, TUM School of Life Sciences, Technical University of Munich, Weihenstephaner Berg 1, 85354 Freising, Germany; caren.tanger@tum.de (C.T.); ulrich.kulozik@tum.de (U.K.)

**Keywords:** pea protein aroma, aldehydes, high-throughput UHPLC-MS/MS, big data analysis, sustainable and innovative food

## Abstract

The aroma of pea protein (*Pisum sativum* L.) was decrypted for knowledge-based flavor optimization of new food products containing pea protein. Sensomics helped to determine several volatiles via ultra-high performance liquid chromatography tandem mass spectrometry and 3-nitrophenylhydrazine derivatization. Among the investigated volatiles, representatives of aldehydes, ketones, and acids were reported in literature as especially important in pea and pea-related matrices. After validation of the method and quantitation of the corresponding analytes, sensory reconstitution as well as omission studies of a selected pea protein were performed and revealed nine odor-active compounds as key food odorants (3-methylbutanal, hexanal, acetaldehyde, (*E,E*)-2,4-nonadienal, (*E*)-2-octenal, benzaldehyde, heptanal, 2-methylbutanal, and nonanoic acid). Interestingly, eight out of nine compounds belonged to the chemical class of aldehydes. Statistical heatmap and cluster analysis of all odor activity values of different pea proteins confirmed the obtained sensory results and generalize these nine key food odorants in other pea proteins. The knowledge of key components gained shows potential for simplifying industrial flavor optimization of pea protein-based food.

## 1. Introduction

In the last several years, an increasing market for alternative foods based on plant ingredients such as pea, soy, oat, or hemp can be observed. While in 2018, the German sales for plant-based dairy substitutes were about €316 million, that number soared by 70% to a total revenue of €536 million in 2020 [1]. Furthermore, in the first quarter of 2020, the production of vegetarian or vegan meat substitutes increased by 37% compared to the previous quarter [2]. Even though these plant-based alternatives are more expensive compared to their original analogs, this does not prevent consumers from buying. This can be explained by an increasing tendency towards sustainability and animal welfare, e.g., as highlighted by a 2020 consumer barometer, saying that 69% of consumers are more willing to pay a higher price for food with sustainable origin [3]. As this current trend apparently results in good business and plant-based proteins while simultaneously showing a better footprint [4], it is indeed linked not only to start-ups but also to well-established companies concentrating their attention and their R&D on this highly topical issue.

Nevertheless, these new foods often suffer from off-flavors introduced by the plant-based ingredients. Aroma perception especially gets distorted by grassy, green, and bean-like changes, as it could be, e.g., shown for different functional milk desserts loaded with microparticulated pea protein as a fat replacer [5]. As Europe offers a large market for pea protein (the second largest in 2016, with 33%) [6], and global rising sales to $285 million through 2026 (compound annual growth rate of 2020–2026: 12%) are predicted [7], the focus of this study is dedicated to pea protein (*Pisum sativum* L.) and its characteristic flavor.

Driven by the aim to lose the distracting pea off-flavor, aroma and taste compositions have been investigated. Activity-guided fractionation in combination with taste dilution experiments could already reveal and explain bitter off-taste in pea protein isolates caused by different lipids and lipid oxidation products [8]. While hexanal and 3-methylbutanoic acid have been unequivocally identified as key food odorants (*KFO*) in raw peas [9], other aroma-active compounds such as methional, 2-undecanone, (*E*)-2-octenal, (*E,E*)-3,5-octadien-2-one, (*E,E*)-2,4-decadienal, or phenylacetaldehyde seem to play a crucial role in pea proteins [10,11]. Nevertheless, a confirmative molecular-sensory aroma reconstitution of pea protein has been missing so far.

Over the last years, the Sensomics concept was successfully adopted to identify many *KFO* in different food matrices, such as in Chinese green tea [12], in Styrian pumpkin seed oil [13], or in raw licorice [14], for instance. By applying the recently described “unified flavor quantitation”, we could advantageously supplement the Sensomics approach by a fast sample preparation, easy handling, and quick UHPLC-MS/MS measurements [15]. In addition, a recently published UHPLC-MS/MS method was also added to cover an increased variety of different odor-active sensometabolites in pea proteins [16].

Commonly reported odorants, identified by GC-O analyses in differently processed pea [9,17], pea proteins [10,11,18], and lupin flours [19], chosen because of their close biological affinity, were also included for targeted quantitation via accurate stable isotope dilution analysis. Therefore, the objective of the present investigation was to decode the aroma of a widely-used pea protein (*Pisum sativum* L.) within Europe, namely Nutralys^®^ S85F, by identifying *KFO* and providing the received knowledge for further flavor optimization, such as downregulation steps. Odor activity calculations followed by sensory reconstitution and omission experiments were performed to achieve an authentic aroma recombinant, as well as to highlight *KFO* in pea protein (*Pisum sativum* L.) for the first time ever.

## 2. Materials and Methods

### 2.1. Chemicals

The following compounds were commercially obtained from Sigma-Aldrich (Steinheim, Germany): 3-nitrophenylhydrazine hydrochloride (3-NPH), pyridine, *N*-(3-(dimethylamino)-propyl)-*N′*-ethylcarbodiimide hydrochloride (EDC), formic acid, triacetin, 3-methylbutanal, hexanal, (*E*)-2-octenal, (*E,E*)-2,4-nonadienal, (*E,Z*)-2,6-nonadienal, (*E,E*)-2,4-decadienal, (*E*)-2-undecenal, (*E*)-2-dodecenal, 2-undecanone, heptanoic acid, phenylacetaldehyde, 4-ethylbenzaldehyde, 4-hydroxy-3-methoxybenzaldehyde (vanillin), *γ*-octalactone, 2,3-dimethylpyrazine, 2,5-dimethylpyrazine, 2,3,5-trimethylpyrazine, 2-ethylpyrazine, 2-ethyl-5(6)-methylpyrazine, 2-isobutyl-3-methoxypyrazine, 3-isopropyl-2-methoxypyrazine, hexanal-*d_12_*, hexanoic acid-*d_3_*, and phenylacetic acid-^13^C_2_. Additionally, 2-methylbutanal was purchased from Alfa Aesar (Lancaster, UK), heptanal from Tokyo Chemcial Industry (Tokyo, Japan), 3-(methylthio)propanal (methional), hexanoic acid, and 2,6-dimethylpyrazine were obtained from Merck KGaA (Darmstadt, Germany), 2,3-octanedione and (*E,E*)-3,5-octadien-2-one from aromaLAB (Planegg, Germany), and 3-methylbutanal-*d_2_* and diacetyl-*d_6_* from CDN Isotopes (Pointe-Claire, QC, Canada). Decanal-*d_2_* [20], vanillin-*d_3_* [21], and *γ*-nonalactone-*d_2_* [22] were synthesized by the Leibniz Institute for Food Systems Biology at the Technical University of Munich. Acetonitrile used for UHPLC-MS/MS analysis was of LC-MS grade (Honeywell, Seelze, Germany). Water for sample preparation and chromatography was purified using a B30 Integrity ultra-pure water system (AQUA LAB GmbH & Co. KG, Ransbach-Baumbach, Germany).

### 2.2. Pea Protein Isolates

The following pea proteins (Table 1) were provided by our partners from the Industrial Collective Research (IGF) branch of the FEI project under grant number AiF 20197 N and analyzed with the developed methods. All samples were stored in the dark at 4 °C.

### 2.3. Protein Content (PC)

Protein contents were determined using the Dumas method with the Vario MAX cube (Elementar Analysensysteme GmbH, Langenselbold, Germany) by the Chair of Food and Bioprocess Engineering at the Technical University of Munich. As proposed for pea proteins, a factor of 5.4 was used for conversion of nitrogen content into protein content [23].

### 2.4. Identification and Quantitation of Odor-Active Acids, Aldehydes, Ketones, and Pyrazines

Internal Standard (IS) solution: First, 3-methylbutanal-*d_2_* (22.0 µg/mL), hexanal-*d_12_* (1.3 µg/mL), decanal-*d_2_* (23.3 µg/mL), diacetyl-*d_6_* (19.9 µg/mL), hexanoic acid-*d_3_* (5.5 µg/mL), phenylacetic acid-^13^C_2_ (2.7 µg/mL), vanillin-*d_3_* (1.8 µg/mL), and *γ*-nonalactone-*d_2_* (15.5 µg/mL) were prepared in acetonitrile/water (50:50, *v*/*v*) and used as an IS mixture for quantitation.

**Figure 1:**
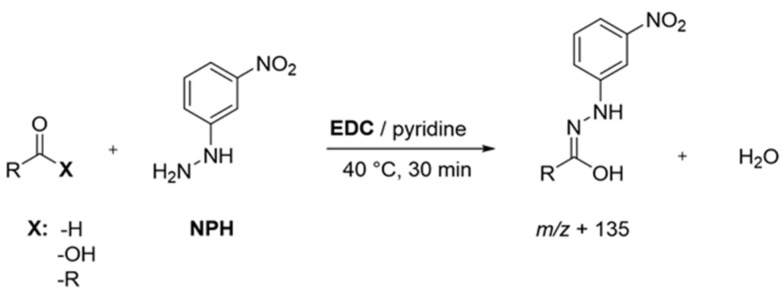
Overview of applied derivatization scheme for the detection of aroma-active compounds. Adapted from [16], with permission from American Chemical Society, 2021.

Sample Preparation: Following literature protocols, with slight modifications to analyze short and branched fatty acids [24] as well as aldehydes, ketones, and organic acids [15,16], protein isolates (40 mg) were first suspended in a mixture of acetonitrile/water (960 µL, 50:50, *v*/*v*), spiked with the IS solution (20 µL), and equilibrated overnight at room temperature under continuous shaking. After at least 20 h, the suspensions were mixed with a solution (20 µL, 200 mmol/L) of 3-NPH in acetonitrile/water (50:50, *v*/*v*) and a solution (20 µL, 120 mmol/L) of EDC in acetonitrile/water (50:50, *v*/*v*) containing 6% pyridine and derivatized for 30 min at 40 °C (Figure 1). A membrane-filtered (Minisart RC 15, 0.45 µm, Sartorius AG, Göttingen, Germany) aliquot (1 µL) was then analyzed via UHPLC-MS/MS (MRM transitions see Table S1).

Screening for pyrazines (Table S2): Pea protein, cocoa, and coffee samples (0.04–1.0 g) were extracted with methanol/water (1–10 mL, 50:50, *v*/*v*) and homogenized for 2 min (Super Homogenizer Precellys Evolution, Bertin Technologies, Montigny-le-Bretonneux, France). Cocoa samples were additionally defatted (*n*-pentane). The centrifugated and membrane-filtered supernatants (1 µL) were then analyzed by UHPLC-MS/MS.

IS calibration curves (Table S3): Stock solutions of 2-methylbutanal (91 µg/mL), 3-methylbutanal (85 µg/mL), hexanal (102 µg/mL), heptanal (105 µg/mL), methional (118 µg/mL), (*E*)-2-octenal (113 µg/mL), (*E,E*)-2,4-nonadienal (116 µg/mL), (*E,Z*)-2,6-nonadienal (124 µg/mL), (*E,E*)-2,4-decadienal (115 µg/mL), (*E*)-2-undecenal (143 µg/mL), (*E*)-2-dodecenal (154 µg/mL), 2,3-octanedione (127 µg/mL), (*E,E*)-3,5-octadien-2-one (97 µg/mL), 2-undecanone (152 µg/mL), hexanoic acid (108 µg/mL), heptanoic acid (112 µg/mL), phenylacetaldehyde (105 µg/mL), 4-ethyl benzaldehyde (164 µg/mL), vanillin (133 µg/mL), and *γ*-octalactone (132 µg/mL) were prepared in acetonitrile/water (50:50, *v*/*v*) and further diluted by one to two steps. Consequently, 16 calibration solutions were produced in acetonitrile/water (50:50, *v*/*v*), and aliquots (40 µL) of each calibration solution spiked with 20 µL of the IS were derivatized as described above. Each determined concentration is the mean of three independent sample workups.

**Figure 2:**
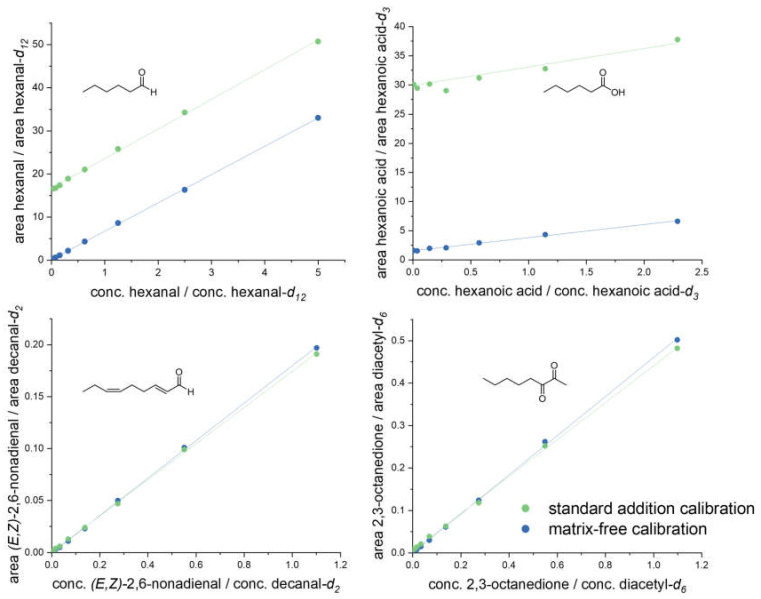
Certain examples (hexanal, hexanoic acid, (*E,Z*)-2,6-nonadienal, 2,3-octanedione) of matrix calibration curves (standard addition calibration) in a suspended pea protein (C) solution, in comparison to matrix-free calibration in acetonitrile/water (50:50, *v*/*v*).

Validation experiments: As no analyte-free pea-like matrix was available for recovery experiments, calibration curves were first analyzed with (standard addition calibration) and without (matrix-free calibration) the presence of pea protein (40 mg/mL, C) in acetonitrile/water (50:50, *v*/*v*), as described above. A comparison between both curves revealed either the same slope, just shifted by a certain amount for the analytes present in pea protein, e.g., for hexanal and hexanoic acid, or congruent curves for no or low abundance, such as for (*E,Z*)-2,6-nonadienal and 2,3-octanedione (Figure 2). Hexanal, hexanoic acid, (*E,Z*)-2,6-nonadienal, and 2,3-octanedione represented one typical compound of each of the investigated compound classes; all analytes indicated the same behavior.

These experiments concluded that matrix effects during ionization were fully compensated by the selected internal standards and, therefore, recovery experiments were recorded to check the extraction and derivatization process in triplicate by spiking a constant volume (20 µL) of the diluted stock solution (1:120) with acetonitrile/water (50:50, *v*/*v*) and equilibrating with the IS solution (20 µL) for at least 20 h. The sample preparation was further conducted as detailed above. For the determination of the limit of detection (LOD) and the limit of quantitation (LOQ), the lowest calibration solution was further diluted, and the signal-to-noise ratio was measured using the MultiQuant software (AB Sciex, Darmstadt, Germany). The LOD was set to a signal-to-noise ratio of 3, and the LOQ was set to a signal-to-noise ratio of 10.

3-NPH-UHPLC-MS/MS analysis: An Exion LC^TM^ UHPLC-system (AB Sciex, Darmstadt, Germany) was connected to a QTRAP 6500^+^ mass spectrometer (AB Sciex) and operated in positive electrospray ionization (ESI^+^) mode (ion spray voltage at +5500 V). The UHPLC system involved two Exion LC AD pumps, an Exion LC degasser, an Exion LC AD autosampler, an Exion LC AC column oven, and an Exion LC controller. Chromatographic separation was achieved on a 100 × 2.1 mm, 100 Å Kinetex 1.7 µm XB-C18 column (Phenomenex, Aschaffenburg, Germany) using the following gradient of 0.1% formic acid in water (solvent A) and 0.1% formic acid in acetonitrile (solvent B) with a flow of 0.4 mL/min: 0 min, 27% B; 0.5 min, 27% B; 1 min, 50% B; 6 min, 100% B; 7 min, 100% B; 7.5 min, 27% B, and 9 min, 27% B. The QTRAP 6500^+^ mass spectrometer was conducted in full-scan mode, as nebulizer (55 psi) and turbo gas (450 °C) zero grade air was used for solvent drying (65 psi). Nitrogen served as curtain (35 psi) and collision gas (1.5 × 10^−5^ torr), and the quadrupoles were set at unit resolution. Data acquisition and instrumental control was performed with the Analyst 1.6.3 software (AB Sciex) and obtained data were evaluated with the MultiQuant software (AB Sciex).

Additional 3-NPH-UHPLC-MS/MS analysis: Beside the described approach, a further 3-NPH-UHPLC-MS/MS method, successfully applied to a model milk dessert [16], was also used for quantitation of further odor-active sensometabolites, for which high abundance among different food matrices was shown [25].

### 2.5. Sensory Analysis

General conditions and panel training: Orthonasal aroma experiments were performed by sixteen aroma panelists (nine women and seven men, age 22–58 years) from the Chair of Food Chemistry and Molecular Sensory Science and the Leibniz Institute for Food Systems Biology at the Technical University of Munich to characterize the aroma profiles of pea proteins (*Pisum sativum* L.). Each attendee trained weekly for a minimum of two years to be able to distinguish aroma qualities and quantities [16]. All panelists agreed to contribute and had no history of known anosmia. For aroma evaluation, aqueous solutions of the following reference odorants (20 mL; 10-fold odor thresholds) for the given odor qualities were used for quantitative descriptive analysis (QDA) training, according to Stone and Sidel [26]: hexanal (25.0 µg/L) for grassy, 3-isopropyl-2-methoxypyrazine (0.1 µg/L) for beans-like, acetic acid (60 mg/L) for sour, (*E,E*)-2,4-decadienal (0.32 µg/L) for fatty, phenylacetic acid (0.68 mg/L) for honey-like, 2-ethyl-5-methylpyrazine (1.0 mg/L) for nutty, 3-methylbutanal (5.0 µg/L) for malty, and 2,3,5-trimethylpyrazine (0.12 mg/L) for earthy. All sensory analyses took place in special sensory cabins, and temperature was regulated to 20–25 °C. Data were evaluated with Excel 2016 (Microsoft, Redmond, WA, USA) as well as Origin 2018b 9.55 (OriginLab Corporation, Northampton, MA, USA).

Aroma profile analysis (APA): For orthonasal aroma profile analysis, 10% pea protein was suspended in a mixture of water/triacetin (97.5:2.5, *w*/*w*) and presented in closed sensory vials (45 mL) to the panelists. By sniffing, the aroma intensities for grassy, beans-like, sour, fatty, honey-like, nutty, malty, and earthy notes were rated from 0 (not detectable) to 5 (very intense), and thus the aroma profiles were characterized.

Recombination studies and comparative APA (cAPA): For aroma recombination, deodorized pea protein powder with strongly minimized characteristic pea aroma was generated using the following steps: Pea protein C (500 g) was stirred in freshly distilled *n*-pentane (2 × 1.5 L), then in dichloromethane (2 × 1.5 L) at room temperature overnight, and was removed from solvent on the next day. The obtained pea protein powder was dried under a stream of nitrogen and could not be sensorially related to pea by the panelists. Moreover, recombination solutions, including all aroma-active compounds (odor activity value, *OAV* ≥ 1) or parts of it (minimal recombinant) in native pea protein concentrations, were prepared in triacetin. Aroma recombinants were prepared by suspending 10% deodorized pea protein powder in water and the recombination solution in triacetin (97.5:2.5, *w*/*w*) and assessed in comparison to C, as described for the APA.

Omission tests: Incomplete recombinants, each lacking one aroma-active compound or group (*OAV* ≥ 1), were evaluated against complete recombinants by means of 3-alternative forced choice tests (3-AFC). Based on the number of correctly identified samples and attended panelists (13–14), *p* values were determined by binomial distribution [27].

### 2.6. Determination of Odor Thresholds

Odor thresholds were taken from the Leibniz-LSB@TUM odorant database or determined in water for 2,3-octanedione, (*E,E*)-3,5-octadien-2-one, and (*E*)-2-dodecenal according to literature [28,29].

### 2.7. Estimation of Aroma Contribution

As only odorants with concentrations achieving their individual threshold contribute to the overall aroma, *OAV* were calculated by using Equation (1). Per definition, analytes with values ≥ 1, determined by the ratio of the quantified amount to the orthonasal odor threshold, had an impact on the food’s authenticity and, thus, were considered in the recombination experiments [25].

(1) $$OAV=\frac{concentration}{orthonasal\;odor\;threshold}$$

### 2.8. Statistical Analysis

*OAV* data were visualized as a heatmap using the visualization platform R (version 4.0.4, R foundation) [30] and package “ComplexHeatmap” [31].

## 3. Results

Based on literature research on the aroma of lupin and pea protein (*Pisum sativum* L.) [9,10,11,17,18,19,32], reported candidates (Tables S1 and S2) and further odor-active sensometabolites [16] were quantitated by means of UHPLC-MS/MS based on stable isotope dilution analysis (SIDA) with rapid and simple sample workup.

### 3.1. Method Development and Validation Experiments

**Figure 3:**
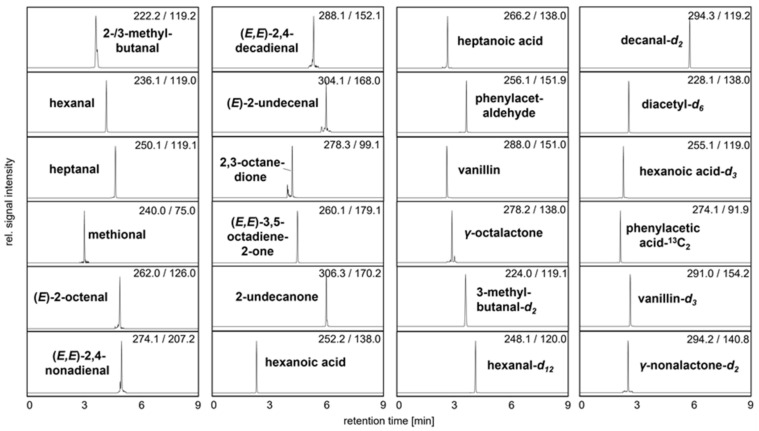
UHPLC-MS/MS analysis showing the mass transitions of quantifiable 3-NPH derivatized analytes and used IS in pea protein C. The signal intensity of each mass transition is normalized.

To guarantee fast, selective, and sensitive quantitation of 3-NPH tagged odorants by means of UHPLC-MS/MS, reference solutions were initially derivatized and used for software-assisted ramping of ion source and ion path parameters by syringe infusion [15,16]. The comparison of retention times and MS spectra could confirm the presence of 2- and 3-methylbutanal, hexanal, heptanal, methional, (*E*)-2-octenal, (*E,E*)-2,4-nonadienal, (*E,Z*)-2,6-nonadienal, (*E,E*)-2,4-decadienal, (*E*)-2-undecenal, (*E*)-2-dodecenal, 2,3-octanedione, (*E,E*)-3,5-octadien-2-one, 2-undecanone, hexanoic acid, heptanoic acid, phenylacetaldehyde, 4-ethyl benzaldehyde, vanillin, and *γ*-octalactone within one single run of nine minutes (Figure 3, for MRM transitions see Table S1).

By establishing scheduled detection windows of ±30 sec, a suspension of 40 mg of protein powders in 1.0 mL acetonitrile/water derivatized with 3-NPH (Figure 1) proved appropriately sensitive to detect all individual analytes [15,16].

Validation experiments revealed no crucial matrix effects for all analytes expressed as parallel upward shifted calibration curves (standard addition calibration), e.g., for hexanal and hexanoic acid (Figure 2). Consequently, recovery rates were calculated by derivatizing known concentrations of each analyte in acetonitrile/water (50:50, *v*/*v*) and analyzing by means of 3-NPH-UHPLC-MS/MS. Determined recovery rates ranged between 80.5 and 106.9%. The LOD and LOQ were very low, varying from <0.1 to 5.8 nmol/L and <0.1 to 19.2 nmol/L, respectively (Table 2). All LOQs showed higher sensitivity than specific aroma thresholds or could be counterbalanced by increasing sample loading, guaranteeing a detection of all analytes to the full extent.

In addition, we attempted to detect the pyrazines 2,3-dimethylpyrazine, 2,5-dimethylpyrazine, 2,6-dimethylpyrazine, 2,3,5-trimethylpyrazine, 2-ethylpyrazine, 2-ethyl-5(6)-methylpyrazine, 3-isopropyl-2-methoxy-(5/6)-methylpyrazine, and 2-isobutyl-3-methoxypyrazine, described in roasted pea and pea protein [9,10,11], using the identical UHPLC-MS/MS method with specific pre-recorded positive (ESI^+^) tuning data of the pyrazines (MRM transitions see Table S2). However, these pyrazines could not be detected in pea protein, even when higher sample amounts were used. In principle, the method could be successfully applied for the identification of pyrazines in coffee and highly roasted cocoa samples. Therefore, we assumed that the pyrazines investigated were below the LOQ and thus they were excluded from further analysis, which is not surprising, as the pea proteins investigated were hardly heat-treated during processing.

Keeping in mind that, of the variety of more than 10,000 different volatiles, less than 3% are primarily responsible for authentic flavor among different food categories [25], a recently published UHPLC-MS/MS flavor method was also used for the analysis of further odor-active sensometabolites [16]: (*Z*)-3-hexenal, (*Z*)-2-nonenal, (*E*)-2-nonenal, *trans*-4,5-epoxy-(*E*)-2-decenal, acetaldehyde, diacetyl, acetoin, 1-hexen-3-one, 1-octen-3-one, acetic acid, butyric acid, 2- and 3-methylbutanoic acid, pentanoic acid, octanoic acid, nonanoic acid, decanoic acid, dodecanoic acid, tetradecanoic acid, phenylacetic acid, benzaldehyde, phenylpropanoic acid, 2-aminoacetophenone, sotolon, 2-acetyl-1-pyrroline, and 2-acetyl-2-thiazoline.

Thus, pea proteins were checked for the presence of the potential aroma-active compounds described in this chapter by UHPLC-MS/MS quantitation methodology.

### 3.2. Sensomics-Assisted Aroma Decoding

Using the Sensomics approach, the aroma composition of pea protein C should be quantitatively decoded and re-engineered. Therefore, in a first step, *OAV* were calculated via the ratio of concentration and corresponding odor threshold in water. This procedure resulted in a list of 27 analytes with *OAV* ≥ 1 (Table 3), at which 3-methylbutanal (5.1 mg/kg; *OAV* 10186), hexanal (14.9 mg/kg; *OAV* 6202), acetaldehyde (72.2 mg/kg; *OAV* 4512), (*E,E*)-2,4-decadienal (101 µg/kg; *OAV* 3741), phenylacetaldehyde (6.1 mg/kg; *OAV* 1173), and (*E,E*)-2,4-nonadienal (53 µg/kg; *OAV* 1157) showed the highest *OAV* > 1000. In contrast, (*E,Z*)-2,6-nonadienal and 4-ethylbenzaldehyde could not be identified in any examined protein sample (Table S4).

Thus far, odorants in pea protein have only once been quantified by means of GC-MS, and relative quantities of several analytes were calculated using hexanal-*d_12_* as an internal standard, yielding in concentrations of 83 mg/kg of hexanal and 2.1 mg/kg of phenylacetaldehyde. (*E,E*)-2,4-decadienal could not be quantitated because of coeluted peaks [10].

Compared to the compounds described above with *OAV* > 1000, the ranges of relative quantities in mg/kg were well in line with those reported in literature [10]. Taking all determined aroma-active compounds (Table 3) into account, reported concentrations for heptanal (16.2 mg/kg), nonanoic acid (4.3 mg/kg), methional (97 µg/kg), 2-undecanone (557 µg/kg), 2,3-octanedione (4.1 mg/kg), and (*E,E*)-3,5-octadien-2-one (24.4 mg/kg) were either slightly higher or, for (*E*)-2-octenal (312 µg/kg), benzaldehyde (6.4 mg/kg), and vanillin (268 µg/kg), marginally lower [10]. Nevertheless, a multitude of different analyte concentrations, such as for 2- and 3-methylbutanoic, pentanoic, hexanoic, heptanoic, octanoic, and dodecanoic acid, could yet not be determined in pea protein.

Based on the results by means of accelerated targeted UHPLC-MS/MS analysis, and on a SIDA using various IS with different functional groups matched on the examined analytes and added before sample preparation, the present quantitation approach provides reliable and precise insights into the quantitative aroma composition of different pea proteins (*Pisum sativum* L.).

### 3.3. Pea Protein Aroma Simulation and Omission Experiments

**Figure 4:**
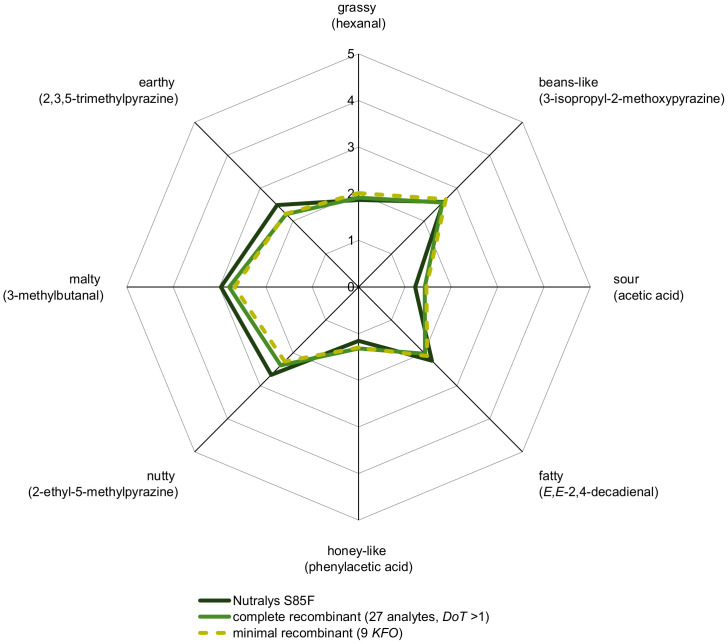
Aroma profile analysis of 10% C in water/triacetin (97.5:2.5, *w*/*w*) and its complete as well as minimal recombinant (10% deodorized protein in water/triacetin, 97.5:2.5, *w*/*w*). Odorants specified in brackets were used as reference attributes for the corresponding odor quality and perceived intensities were rated from 0 (not detectable) to 5 (very intense) by the panelists.

By means of *cAPA*, a complete aroma recombinant, consisting of all 27 analytes with *OAV* > 1 (Table 3) in native concentrations in a deodorized pea protein solution, was evaluated sensorially in comparison to C (Figure 4). The results proved the quantified aroma compounds in their correct ratio and the successful aroma reconstitution, respectively.

In a next step, aroma compounds were omitted and evaluated sensorially via 3-AFC tests in order to highlight which of the odorants were *KFO* and to assess the individual impact of singly omitted odorants (tests O3–O15) or blocks (O1 and O2) (Table 4) on the overall aroma.

The first experiments revealed that an omission of all aroma-active compounds with *OAV* < 10 (test O1) did not lead to a significant difference (*p* > 5%), indicating minor importance of hexanoic, octanoic, phenylacetic, and 2-methylbutanoic acids as well as *γ*-octalactone, 2,3-octanedione, 2-undecanone, acetoin, and (*E,E*)-3,5-octadien-2-one for the overall aroma of pea protein C. In addition, the omission of the group of odorants with *OAV* < 100 (test O2: methional, vanillin, acetic, 3-methylbutanoic, and decanoic acids) could not be significantly (*p* > 5%) distinguished by the panel. For *OAV* > 100, all 13 odorants were individually omitted and tested against the complete recombinants, whereupon two odorants, namely 3-methylbutanal (*OAV* 10186, test O3) and heptanal (*OAV* 217, test O12), led to very highly significant differences (*p* < 0.1%). The omission of 2-methylbutanal (*OAV* 178, test O13) caused a highly significant difference (1% ≥ *p* < 0.1%), while omissions of hexanal (*OAV* 6202, test O4), acetaldehyde (*OAV* 4512, test O5), (*E,E*)-2,4-nonadienal (*OAV* 1157, test O8), (*E*)-2-octenal (*OAV* 533, test O9), benzaldehyde (*OAV* 248, test O11), and nonanoic acid (*OAV* 107, test O15) still led to significant differences (5% ≥ *p* > 1%). In contrast, omissions of (*E,E*)-2,4-decadienal (*OAV* 3741, test O6), phenylacetaldehyde (*OAV* 1173, test O7), and diacetyl (*OAV* 329, test O10) as well as (*E*)-2-undecenal (OAV 158, test O14) resulted in no significant differences (*p* > 5%) and, consequently, together with the odorants of *OAV* < 100, were considered as no *KFO*. Finally, a minimal recombinant based on these nine analytes (Figure 4) highlighted very high similarity compared to the complete recombinant consisting of 27 odorants with *OAV* > 1 (Table 3) and is underlining once more their role as *KFO* and importance on the overall aroma of pea protein C.

### 3.4. OAV Mapping of Commercially Available Pea Proteins

**Figure 5:**
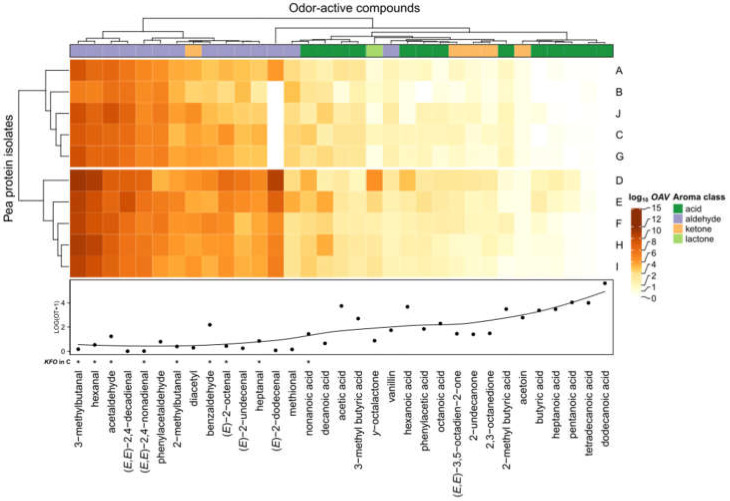
*OAV* mapping (heatmap) based on quantifiable odorants in different pea protein samples (A–J) and the corresponding odor thresholds (OT) determined in water. *OAV* data are log transformed. Asterisk (*) indicates *KFO* carried out with pea protein C by means of the Sensomics concept.

As aroma re-engineering and discovery of *KFO* by means of the Sensomics approach is complex and time-consuming, the question arose whether the impact of revealed *KFO* in pea protein C was also transferrable to the overall aroma of pea proteins (*Pisum sativum* L.) in general. Therefore, further pea protein isolates (A, B, D, E, F, G, H, I, J) were also analyzed with the described UHPLC-MS/MS methods and checked for molecular sensory differences. To be able to draw reliable conclusions, *OAVs* were calculated (Table S5) and logarithmically visualized based on the quantified concentrations and odor thresholds of each analyte in the examined pea protein samples (Figure 5).

The first insights indicated that mainly aldehydes with relatively low odor thresholds automatically clustered together, underlining the importance on the overall aroma among all pea proteins. Moreover, eight out of nine *KFO* could be spotted within this cluster, namely 3-methylbutanal, hexanal, acetaldehyde, (*E,E*)-2,4-nonadienal, 2-methylbutanal, benzaldehyde, (*E*)-2-octenal, and heptanal.

Except for nonanoic acid, also determined as *KFO* in C, and diacetyl, further acids, ketones, and *γ*-octalactone seemed to play a minor role for the aroma. Thus, statistical cluster analysis additionally substantiated the sensory results obtained by omission experiments.

Furthermore, cluster analysis also revealed intra-pea protein variations expressed by two different protein groups: A, B, J, C, G (cluster 1) and D, E, F, H, I (cluster 2). While, e.g., (*E*)-2-dodecenal was highly present in cluster 2, it did not exceed the *OAV* > 1 for pea proteins within cluster 1. Additionally, the individual contribution of *KFO* aldehydes slightly differed among the examined pea proteins.

In summary, *OAV* heatmapping highlighted comparable influences on the overall aroma of pea proteins and emphasized the group of aldehydes. Moreover, there were strong indications that the examined *KFO* of pea protein C were transferable as general *KFO* among different pea proteins (*Pisum sativum* L.).

## 4. Conclusions

**Figure 6:**
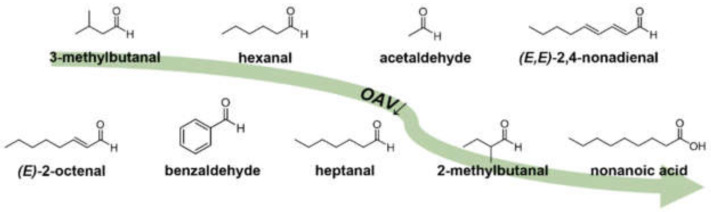
Chemical structures of *KFO* in pea protein (*Pisum sativum* L.) in descending *OAV* order, revealed by means of 3-AFC omission and *OAV* heatmapping.

Pea protein (*Pisum sativum* L.) could be a promising ingredient for the development of new functional foods such as meat substitutes. Unfortunately, the characteristic grassy, green, and bean-like off-flavor often distracts consumers, whereby these foods suffer from a lower acceptance and consequently need to be optimized regarding flavor. By means of high-throughput UHPLC-MS/MS analysis including 3-NPH derivatization, the presence and concentration of selected odorants, described to be important in different pea or related matrices, could be evaluated within a few minutes. *OAV* of the quantified analytes revealed 27 odor-active compounds, which resulted in their distinctive concentration composition in a confirmative aroma recombination. Finally, recombination and omission experiments as well as *OAV* heatmapping highlighted nine general key food odorants in pea protein, namely, 3-methylbutanal, hexanal, acetaldehyde, (*E,E*)-2,4-nonadienal, (*E*)-2-octenal, benzaldehyde, heptanal, 2-methylbutanal, and nonanoic acid (Figure 6).

The present investigation provides new insights into the aroma of different pea proteins (*Pisum sativum* L.) by *KFO* identification as well as cluster analysis and, therefore, may be helpful for knowledge-based flavor optimization of foods using pea protein as a (main) ingredient.

## Figures and Tables

**Table 1:** List of analyzed pea proteins within FEI project AiF 20197 N.

Code	Commercial Name	PC ^(a)^	Batch	Producer
A	Nutralys F85F	67%	W084M	Roquette, Lestrem, France
B	Prestige	42%	KO67X	Parrheim Foods, Saskatoon, Canada
C	Nutralys S85F	68%	W317M	Roquette, Lestrem, France
D	Bio Erbsen Protein	70%	15720-30118	Golden Peanut, Garstedt, Germany
E	Bio Erbsen Protein	68%	170180323	Piowald, Mühbrook, Germany
F	1501018	68%	83526272	Döhler, Darmstadt, Germany
G	Pea Pro	68%	06102016	LSP Sports Nutrition, Bonn, Germany
H	Empro E86HV	72%	41266	Emsland-Stärke, Emlichheim, Germany
I	Empro E86	71%	41266	Emsland-Stärke, Emlichheim, Germany
J	Pisane C9	69%	817021	Cosucra Group, Warcoing, Belgium

^(a)^ PC = Protein Content, determined using the Dumas method and a conversion factor of 5.4 (see Section 2.3).

**Table 2:** Performed validation experiments of important odorants in pea protein isolates.

Validated Analyte	Add. ^(a)^ (µmol/L)	Add. Found ± SD ^(b)^ (µmol/L)	RSD ^(c)^ (%)	Recovery (%)	LOD ^(d)^ (nmol/L)	LOQ ^(e)^ (nmol/L)
2-/3-methylbutanal, sum	0.668	0.667 ± 0.038	5.7	99.8	<0.1	<0.1
2-methylbutanal	0.346	0.344 ± 0.009	2.7	99.5	<0.1	<0.1
hexanal	0.333	0.277 ± 0.004	1.5	83.0	2.7	9.0
heptanal	0.301	0.301 ± 0.006	1.8	99.8	<0.1	<0.1
methional	0.370	0.336 ± 0.008	2.4	90.8	0.2	0.5
(*E*)-2-octenal	0.292	0.259 ± 0.003	1.0	88.8	0.5	1.7
(*E,E*)-2,4-nonadienal	0.274	0.272 ± 0.004	1.5	99.3	0.2	0.5
(*E,Z*)-2,6-nonadienal	0.294	0.294 ± 0.008	2.8	100.0	0.2	0.8
(*E,E*)-2,4-decadienal	0.246	0.241 ± 0.003	1.3	98.0	0.2	0.5
(*E*)-2-undecenal	0.278	0.267 ± 0.020	7.4	96.2	0.3	0.9
(*E*)-2-dodecenal	0.275	0.258 ± 0.015	5.7	93.7	1.4	4.7
2,3-octanedione	0.293	0.290 ± 0.003	0.9	99.0	0.4	1.2
(*E,E*)-3,5-octadien-2-one	0.256	0.264 ± 0.006	2.1	103.2	<0.1	0.3
2-undecanone	0.291	0.288 ± 0.011	3.8	99.1	<0.1	<0.1
hexanoic acid	0.305	0.288 ± 0.012	4.3	94.4	<0.1	<0.1
heptanoic acid	0.280	0.285 ± 0.013	4.7	101.7	5.8	19.2
phenylacetaldehyde	0.286	0.306 ± 0.009	2.9	106.9	<0.1	0.3
4-ethyl benzaldehyde	0.400	0.378 ± 0.023	6.1	94.5	4.7	15.8
vanillin	0.285	0.258 ± 0.012	4.7	90.6	0.2	0.6
*γ*-octalactone	0.302	0.243 ± 0.010	4.0	80.5	1.0	3.2

^(a)^ Add. = Addition. ^(b)^ Add. found = Addition found, SD = Standard Deviation, determined based on replicate sample workup and analysis (*n* = 3). ^(c)^ RSD = Relative Standard Deviation. ^(d)^ LOD = Limit of Detection, determined based on a signal-to-noise ratio of 3. ^(e)^ LOQ = Limit of Quantitation, determined based on a signal-to-noise ratio of 10.

**Table 3:** Concentrations of aroma-active compounds in pea protein C in descending *OAV* order.

No.	Aroma-Active Analytes	Used IS	Odor Quality	Mean ± SD ^(a)^ (µg/kg)	RSD ^(b)^ (%)	OT ^(c)^ (µg/kg)	*OAV* ^(d)^
**1**	3-methylbutanal ^(e)^	3-methylbutanal-*d_2_*	malty	5093 (5360 ± 547) ^(e)^	(10.2) ^(e)^	0.5	10186
**2**	hexanal	hexanal-*d_12_*	green, grassy	14,886 ± 1904	12.8	2.4	6202
**3**	acetaldehyde ^(f)^	acetaldehyde-*d_3_*	fresh, green	72,197 ± 4231	5.9	16	4512
**4**	(*E,E*)-2,4-decadienal	decanal-*d_2_*	fatty, deep-fried	101 ± 10	9.8	0.027	3736
**5**	phenylacetaldehyde	phenylacetic acid-^13^C_2_	flowery, honey-like	6097 ± 303	5.0	5.2	1173
**6**	(*E,E*)-2,4-nonadienal	decanal-*d_2_*	fatty, green	53 ± 7	12.5	0.046	1156
**7**	(*E*)-2-octenal	hexanal-*d_12_*	fatty, nutty	907 ± 8	0.8	1.7	533
**8**	diacetyl ^(f)^	diacetyl-*d_6_*	butter-like	316 ± 25	8.0	0.96	329
**9**	benzaldehyde ^(f)^	phenylacetic acid-^13^C_2_	bitter almond-like, marzipan-like	37,201 ± 7850	21.1	150	248
**10**	heptanal	hexanal-*d_12_*	citrus-like, fatty	1326 ± 130	9.8	6.1	217
**11**	2-methylbutanal	3-methylbutanal-*d_2_*	malty	267 ± 8	2.9	1.5	178
**12**	(*E*)-2-undecenal	decanal-*d_2_*	soapy, metallic	123 ± 8	6.5	0.78	157
**13**	nonanoic acid ^(f)^	octanoic acid-*d_15_*	moldy, pungent	2776 ± 167	6.0	26	107
**14**	methional	hexanal-*d_12_*	cooked potato-like	37 ± 5	4.7	0.43	85
**15**	acetic acid ^(f)^	acetic acid-^13^C_2_	vinegar-like	262,037 ± 10,440	4.0	5600	47
**16**	3-methylbutanoic acid ^(f)^	butyric acid-^13^C_4_	sweaty	23,274 ± 1425	6.1	490	47
**17**	decanoic acid ^(f)^	octanoic acid-*d_15_*	soapy, musty	51 ± 3	5.5	3.5	15
**18**	vanillin	vanillin-*d_3_*	vanilla-like, sweet	561 ± 18	3.2	53	11
**19**	(*E,E*)-3,5-octadien-2-one	diacetyl-*d_6_*	woody, mushroom-like, green	231 ± 18	7.9	27	9
**20**	hexanoic acid	hexanoic acid-*d_3_*	sweaty	36,802 ± 1978	5.4	4800	8
**21**	octanoic acid ^(f)^	octanoic acid-*d_15_*	carrot-like, musty	1536 ± 48	3.1	190	8
**22**	phenylacetic acid ^(f)^	phenylacetic acid-^13^C_2_	honey-like, beeswax-like	516 ± 61	11.8	68	8
**23**	*γ*-octalactone	*γ*-nonalactone-*d_2_*	coconut-like	47 ± 5	10.5	6.5	7
**24**	2-methylbutanoic acid ^(f)^	butyric acid-^13^C_4_	malty, fruity, sweaty	21,419 ± 1014	4.7	3100	7
**25**	2,3-octanedione	diacetyl-*d_6_*	mushroom-like, dill-like, broccoli-like	141 ± 13	8.9	29	4.8
**26**	2-undecanone	decanal-*d_2_*	soapy, green	68 ± 4	6.5	24	2.8
**27**	acetoin ^(f)^	diacetyl-*d_6_*	butter-like, carrot-like	978 ± 7	0.7	590	1.7

^(a)^ Mean = arithmetic mean, SD = Standard Deviation, determined based on replicate sample workup and analysis (*n* = 3). ^(b)^ RSD = Relative Standard Deviation. ^(c)^ OT = Odor Threshold in water, taken from the Leibniz-LSB@TUM odorant database [29]. ^(d)^ Concentration divided by the odor threshold and expressed as *OAV* (Odor Activity Value). ^(e)^ The concentration of 3-methylbutanal was determined by subtraction the sum value (in brackets) from the 2-methylbutanal concentration. ^(f)^ Quantified by an additional 3-NPH-UHPLC-MS/MS method published for dairy analysis [16].

**Table 4:** Omission experiments applied to the aroma model of pea protein C.

Test	Odorant(s) Omitted ^(a)^	*OAV*	*p* Value (%)	Significance ^(b)^
O1	**19–27**	<10	23.8	NS
O2	**14–18**	<100	19.1	NS
O3	**1**, 3-methylbutanal	10186	<0.1	***
O4	**2**, hexanal	6202	2.6	*
O5	**3**, acetaldehyde	4512	4.8	*
O6	**4**, (*E,E*)-2,4-decadienal	3736	9.2	NS
O7	**5**, phenylacetaldehyde	1173	21.4	NS
O8	**6**, (*E,E*)-2,4-nonadienal	1156	4.8	*
O9	**7**, (*E*)-2-octenal	533	1.3	*
O10	**8**, diacetyl	329	15.6	NS
O11	**9**, benzaldehyde	248	4.0	*
O12	**10**, heptanal	217	<0.1	***
O13	**11**, 2-methylbutanal	178	0.7	**
O14	**12**, (*E*)-2-undecenal	157	23.0	NS
O15	**13**, nonanoic acid	107	4.0	*

^(a)^ Odorant numbers refer to Table 3. ^(b)^ NS, no significance (*p* > 5%); *, significance (5% ≥ *p* > 1%); **, highly significance (1% ≥ *p* > 0.1%); ***, very highly significance (*p* ≤ 0.1%).

## Data Availability

All data used in this publication are saved at the Chair for Food Chemistry and Molecular Sensory Science, Freising, Germany.

## Supplementary Materials

The following are available online at https://www.mdpi.com/article/10.3390/foods11030412/s1, Table S1: MRM transitions of analyzed 3-NPH tagged odorants, Table S2: MRM transitions of pyrazines, Table S3: Used IS, IS calibration curves, and R2 of the quantified odorants, Table S4: Concentrations of the quantified odorants in different pea protein samples, Table S5: OAV of the quantified odorants in different pea protein samples and R script of OAV heatmap.
