# Supplementary material for: Sensomics-Assisted Aroma Decoding of Pea Protein Isolates (Pisum sativum L.)
Source: Foods. 2022 Jan 30;11(3):412. doi: 10.3390/foods11030412 (PMC8834122; doi:10.3390/foods11030412)
Supplement: Supplementary file 1 [file foods-11-00412-s001.zip › foods-1571349-supplementary.pdf]

Supporting Information

Sensomics-assisted aroma decoding of pea protein isolates (*Pisum sativum* L.)

Florian Utz,[1] Andrea Spaccasassi,[1] Johanna Kreissl,[2] Timo D. Stark,[1] Caren Tanger,[3] Ulrich Kulozik,[3] Thomas Hofmann,[1] and Corinna Dawid[1*]

[1] Chair of Food Chemistry and Molecular Sensory Science, Technical University of Munich, Lise-Meitner-Straße 34, 85354 Freising, Germany,

[2] Leibniz-Institute for Food Systems Biology at the Technical University of Munich, Lise-Meitner-Straße 34, 85354 Freising, Germany, and

[3] Chair of Food and Bioprocess Engineering, Technical University of Munich, Weihenstephaner Berg 1, 85354 Freising, Germany

---

[*] **Author to whom correspondence should be addressed**

PHONE +49-8161/71-2902

FAX +49-8161/71-2949

E-MAIL corinna.dawid@tum.de

Table S1. MRM transitions of analyzed 3-NPH tagged odorants.

Analyte (number) *qt.*/*ql.*	Ionization	Q1 [*m/z*]	Q3 [*m/z*]	t_R [min]	DP [V]	EP [V]	CE [V]	CXP [V]
2-methylbutanal *qt*.	ESI^+	222.1	152.0	3.7	46	10	15	18
2-methylbutanal *ql.*	ESI^+	222.1	118.9	3.7	46	10	19	6
3-methylbutanal *qt*.	ESI^+	221.9	119.2	3.6	36	10	19	12
3-methylbutanal *ql.*	ESI^+	221.9	137.1	3.6	36	10	21	8
(IS) 3-methylbutanal-d_2 *qt*.	ESI^+	224.0	119.1	3.6	1	10	21	14
(IS) 3-methylbutanal-d_2 *ql.*	ESI^+	224.0	137.0	3.6	1	10	23	16
hexanal *qt*.	ESI^+	236.1	119.0	4.2	46	10	19	14
hexanal *ql.*	ESI^+	236.1	137.0	4.2	46	10	23	14
(IS) hexanal-d_{12} *qt.*	ESI^+	248.1	120.0	4.1	41	10	21	14
(IS) hexanal-d_{12} *ql.*	ESI^+	248.1	137.0	4.1	41	10	23	16
heptanal *qt.*	ESI^+	250.1	119.1	4.6	71	10	21	16
heptanal *ql.*	ESI^+	250.1	136.9	4.6	71	10	25	18
methional *qt*.	ESI^+	240.0	75.0	3.0	6	10	17	8
methional *ql.*	ESI^+	240.0	139.0	3.0	6	10	11	14
(*E*)-2-octenal *qt.*	ESI^+	262.0	126.0	4.9	121	10	23	14
(*E*)-2-octenal *ql.*	ESI^+	262.0	137.0	4.9	121	10	25	16
(*E*,*E*)-2,4-nonadienal *qt.*	ESI^+	274.1	207.2	5.0	1	10	15	12
(*E*,*E*)-2,4-nonadienal *ql.*	ESI^+	274.1	152.0	5.0	1	10	21	16
(*E*,*Z*)-2,6-nonadienal *qt.*	ESI^+	274.1	119.2	4.8	1	10	27	12
(*E*,*Z*)-2,6-nonadienal *ql.*	ESI^+	274.1	138.2	4.8	1	10	19	10
(*E*,*E*)-2,4-decadienal *qt.*	ESI^+	288.1	152.1	5.3	1	10	25	20
(*E*,*E*)-2,4-decadienal *ql.*	ESI^+	288.1	221.1	5.3	1	10	17	14
(*E*)-2-undecenal *qt.*	ESI^+	304.1	168.0	6.0	1	10	27	20
(*E*)-2-undecenal *ql.*	ESI^+	304.1	118.9	6.0	1	10	31	20
(*E*)-2-dodecenal *qt.*	ESI^+	318.2	182.2	6.3	1	10	27	8
(*E*)-2-dodecenal *ql.*	ESI^+	318.2	271.1	6.3	1	10	33	16
(IS) decanal-d_2 *qt.*	ESI^+	294.3	119.2	5.7	1	10	23	20
(IS) decanal-d_2 *ql.*	ESI^+	294.3	158.2	5.7	1	10	23	10
2,3-octanedione *qt.*	ESI^+	278.3	99.1	4.2	1	10	13	12
2,3-octanedione *ql.*	ESI^+	278.3	71.1	4.2	1	10	17	8
(*E*,*E*)-3,5-octadien-2-one *qt.*	ESI^+	260.1	179.1	4.5	76	10	15	6

(*E,E*)-3,5-octadien-2-one *ql.*	ESI^+	260.1	124.1	4.5	76	10	25	10
(IS) diacetyl-d_6 *qt.*	ESI^+	228.1	138.0	2.5	41	10	23	18
(IS) diacetyl-d_6 *ql.*	ESI^+	228.1	91.9	2.5	41	10	39	14
2-undecanone *qt.*	ESI^+	306.3	170.2	6.0	1	10	31	10
2-undecanone *ql.*	ESI^+	306.3	133.1	6.0	1	10	29	14
hexanoic acid *qt.*	ESI^+	252.2	138.0	2.3	1	10	23	16
hexanoic acid *ql.*	ESI^+	252.2	116.1	2.3	1	10	23	12
(IS) hexanoic acid-d_3 *qt.*	ESI^+	255.1	119.0	2.3	81	10	25	14
(IS) hexanoic acid-d_3 *ql.*	ESI^+	255.1	137.8	2.3	81	10	23	18
heptanoic acid *qt.*	ESI^+	266.2	138.0	2.6	156	10	23	16
heptanoic acid *ql.*	ESI^+	266.2	130.1	2.6	156	10	23	16
phenylacetaldehyde *qt.*	ESI^+	256.1	151.9	3.6	61	10	19	18
phenylacetaldehyde *ql.*	ESI^+	256.1	118.9	3.6	61	10	21	14
(IS) phenylacetic acid-$^{13}C_2$ *qt.*	ESI^+	274.1	91.9	2.1	76	10	23	10
(IS) phenylacetic acid-$^{13}C_2$ *ql.*	ESI^+	274.1	138.1	2.1	76	10	23	16
4-ethyl benzaldehyde *qt.*	ESI^+	270.2	134.1	4.3	101	10	25	16
4-ethyl benzaldehyde *ql.*	ESI^+	270.2	223.1	4.3	101	10	23	26
vanillin *qt.*	ESI^+	288.0	151.0	2.6	1	10	21	18
vanillin *ql.*	ESI^+	288.0	105.0	2.6	1	10	35	12
(IS) vanillin-d_3 *qt.*	ESI^+	291.0	154.2	2.6	41	10	27	18
(IS) vanillin-d_3 *ql.*	ESI^+	291.0	108.9	2.6	41	10	55	12
γ-octalactone *qt.*	ESI^+	278.2	138.0	2.9	106	10	27	16
γ-octalactone *ql.*	ESI^+	278.2	92.0	2.9	106	10	47	14
(IS) *γ*-nonalactone-d_2 *qt.*	ESI^+	294.2	140.8	2.5	106	10	9	16
(IS) *γ*-nonalactone-d_2 *ql.*	ESI^+	294.2	69.9	2.5	106	10	19	8

qt. = quantifier MRM. ***ql.*** = qualifier MRM. **Q1** = precursor ion. **Q3** = selected product ion. $\mathbf{t_R}$ = retention time. **DP** = declustering potential. **EP** = entrance potential. **CE** = collision energy. **CXP** = cell exit potential.

Table S2. MRM transitions of pyrazines.

Analyte (number) *qt./ql.*	Ionization	Q1 [*m/z*]	Q3 [*m/z*]	t_R [min]	DP [V]	EP [V]	CE [V]	CXP [V]
2,3-dimethylpyrazine *qt.*	ESI^+	108.7	68.2	0.8	1	10	25	10
2,3-dimethylpyrazine *ql.*	ESI^+	108.7	42.2	0.8	1	10	43	18
2,5-dimethylpyrazine *qt.*	ESI^+	108.9	82.2	0.8	21	10	25	10
2,5-dimethylpyrazine *ql.*	ESI^+	108.9	41.2	0.8	21	10	33	18
2,6-dimethylpyrazine *qt.*	ESI^+	108.8	68.0	0.8	71	10	25	8
2,6-dimethylpyrazine *ql.*	ESI^+	108.8	39.1	0.8	71	10	49	16
2,3,5-trimethylpyrazine *qt.*	ESI^+	122.9	82.2	0.9	1	10	25	6
2,3,5-trimethylpyrazine *ql.*	ESI^+	122.9	55.0	0.9	1	10	29	14
2-ethylpyrazine *qt.*	ESI^+	108.8	82.1	1.0	26	10	25	10
2-ethylpyrazine *ql.*	ESI^+	108.8	67.1	1.0	26	10	35	18
2-ethyl-5(6)-methylpyrazine *qt.*	ESI^+	123.2	79.9	1.2	11	10	39	16
2-ethyl-5(6)-methylpyrazine *ql.*	ESI^+	123.2	81.1	1.2	11	10	35	38
2-isobutyl-3-methoxypyrazine *qt.*	ESI^+	167.3	94.0	5.2	1	10	37	10
2-isobutyl-3-methoxypyrazine *ql.*	ESI^+	167.3	95.0	5.2	1	10	37	10

qt. = quantifier MRM. ***ql.*** = qualifier MRM. **Q1** = precursor ion. **Q3** = selected product ion. $\mathbf{t_R}$ = retention time. **DP** = declustering potential. **EP** = entrance potential. **CE** = collision energy. **CXP** = cell exit potential.

Table S3. Used isotopically labeled internal standards (IS), IS calibration curves and coefficients of determination (R^2) of the quantified odorants.

odorants	IS	calibration curve	R^2
2-/3-methylbutanal sum	3-methylbutanal-d_2	y = 0.19155x + 0.03619	0.99854
hexanal	hexanal-d_{12}	y = 9.13467x + 0.98183	0.99994
acetaldehyde	acetaldehyde-d_3	y = 0.00581x + 0.00752	0.99990
(*E,E*)-2,4-decadienal	decanal-d_2	y = 0.39907x + 0.00008	0.99996
phenylacetaldehyde	phenylacetic acid-$^{13}C_2$	y = 1.15706x + 0.00644	0.99953
(*E,E*)-2,4-nonadienal	decanal-d_2	y = 0.48497x − 0.00228	0.99990
(*E,Z*)-2,6-nonadienal	decanal-d_2	y = 0.16550x + 0.00081	0.99992
(*E*)-2-octenal	hexanal-d_{12}	y = 0.66803x − 0.00720	0.99983
diacetyl	diacetyl-d_6	y = 0.30896x + 0.01021	0.99766
benzaldehyde	phenylacetic acid-$^{13}C_2$	y = 2.64386x + 0.02578	0.99990
4-ethyl benzaldehyde	phenylacetic acid-$^{13}C_2$	y = 4.60971x − 0.06686	0.99921
heptanal	hexanal-d_{12}	y = 5.42371x − 0.00338	0.99948
2-methylbutanal	3-methylbutanal-d_2	y = 0.22279x – 0.01637	0.99851
(*E*)-2-undecenal	decanal-d_2	y = 0.20685x − 0.00133	0.99975
nonanoic acid	octanoic acid-d_{15}	y = 4.11993x + 0.56915	0.99791
methional	hexanal-d_{12}	y = 0.83702x − 0.00694	0.99996
acetic acid	acetic acid-$^{13}C_2$	y = 0.48454x + 1.77261	0.99876
3-methylbutanoic acid	butyric acid-$^{13}C_4$	y = 1.95382x + 0.15773	0.99967
decanoic acid	octanoic acid-d_{15}	y = 3.21178x + 0.35795	0.99971
vanillin	vanillin-d_3	y = 14.64794x + 0.09213	0.99990
(*E,E*)-3,5-octadien-2-one	diacetyl-d_6	y = 4.86112x − 0.01880	0.99879
hexanoic acid	hexanoic acid-d_3	y = 3.05442x + 0.78237	0.99890
octanoic acid	octanoic acid-d_{15}	y = 1.89253x + 0.26281	0.99941
phenylacetic acid	phenylacetic acid-$^{13}C_2$	y = 5.36691x + 0.03406	0.99978
γ-octalactone	*γ*-nonalactone-d_2	y = 2.76661x + 0.00986	0.99990
2-methylbutanoic acid	butyric acid-$^{13}C_4$	y = 0.89721x − 0.00920	0.99975
2,3-octanedione	diacetyl-d_6	y = 0.80316x − 0.00268	0.99967
2-undecanone	decanal-d_2	y = 4.57128x − 0.04016	0.99955
(*E*)-2-dodecenal	decanal-d_2	y = 0.21231x − 0.00085	0.99988
acetoin	diacetyl-d_6	y = 1.23169x − 0.52823	0.99310
butyric acid	butyric acid-$^{13}C_4$	y = 1.14743x + 0.19934	0.99965
pentanoic acid	butyric acid-$^{13}C_4$	y = 1.55311x + 0.13216	0.99991
heptanoic acid	hexanoic acid-d_3	y = 0.38546x + 0.02620	0.99981
dodecanoic acid	decanoic acid-^{13}C	y = 0.33867x + 0.04795	0.99936
tetradecanoic acid	decanoic acid-^{13}C	y = 0.37087x + 0.05286	0.99781

Table S4. Concentrations in mg/kg (±RSD) of the quantified odorants in different pea protein samples by means of 3-NPH-UHPLC-MS/MS.

odorants	A	B	C	D	E	F	G	H	I	J
3-methylbutanal	7.1	0.8	5.1	43.5	21.9	19.0	9.1	29.1	19.2	9.4
hexanal	10.2 (±7.8%)	3.0 (±4.2%)	14.9 (±12.8%)	138.0 (±2.9%)	43.4 (±8.2%)	33.9 (±5.8%)	11.6 (±8.6%)	83.4 (±3.5%)	43.3 (±7.4%)	14.2 (±10.6%)
acetaldehyde	79.7 (±8.8%)	47.5 (±5.3%)	72.2 (±5.9%)	131.5 (±3.0%)	80.5 (±4.2%)	158.4 (±3.1%)	82.5 (±4.9%)	117.2 (±6.9%)	93.2 (±11.0%)	253.6 (±13.0%)
(*E*,*E*)-2,4-decadienal	0.1 (±4.9%)	0.1 (±7.2%)	0.1 (±9.7%)	0.2 (±6.9%)	0.5 (±7.7%)	0.1 (±1.5%)	0.1 (±4.0%)	0.1 (±7.6%)	0.1 (±9.7%)	0.1 (±2.1%)
phenylacetaldehyde	3.1 (±12.1%)	5.9 (±2.2%)	6.1 (±5.0%)	1.2 (±3.9%)	8.8 (±10.3%)	10.6 (±2.3%)	7.1 (±11.9%)	5.9 (±6.4%)	2.0 (±5.3%)	9.6 (±11.7%)
(*E*,*E*)-2,4-nonadienal	0.04 (±3.9%)	0.04 (±7.9%)	0.1 (±12.5%)	0.2 (±4.7%)	0.1 (±9.8%)	0.1 (±8.6%)	0.04 (±3.0%)	0.1 (±6.5%)	0.1 (±8.7%)	0.04 (±3.4%)
(*E*,*Z*)-2,6-nonadienal	nd	nd	nd	nd	nd	nd	nd	nd	nd	nd
(*E*)-2-octenal	0.3 (±7.4%)	0.1 (±14.8%)	0.9 (±0.8%)	6.8 (±1.9%)	2.0 (±4.6%)	1.3 (±8.7%)	0.4 (±5.7%)	0.8 (±8.7%)	0.9 (±4.9%)	0.2 (±12.9%)
diacetyl	0.2 (±6.0%)	0.1 (±1.0%)	0.3 (±8.0%)	0.5 (±5.5%)	0.3 (±9.8%)	0.5 (±7.1%)	0.4 (±5.3%)	0.3 (±8.1%)	0.5 (±9.3%)	0.7 (±6.9%)
benzaldehyde	20.3 (±9.4%)	15.2 (±9.6%)	37.2 (±21.1%)	142.8 (±10.6%)	36.1 (±1.9%)	146.9 (±11.9%)	48.8 (±8.5%)	59.8 (±12.1%)	35.5 (±10.5%)	26.2 (±8.5%)
4-ethyl benzaldehyde	nd	nd	nd	nd	nd	nd	nd	nd	nd	nd
heptanal	0.7 (±5.5%)	0.1 (±2.8%)	1.3 (±9.8%)	5.8 (±14.2%)	2.8 (±13.5%)	1.7 (±3.7%)	0.8 (±3.1%)	1.9 (±10.3%)	1.7 (±15.4%)	0.6 (±4.6%)
2-methylbutanal	0.4 (±5.4%)	0.3 (±6.4%)	0.3 (±2.9%)	0.5 (±0.8%)	1.5 (±5.6%)	0.8 (±4.1%)	0.4 (±2.9%)	0.6 (±4.6%)	0.4 (±6.4%)	0.5 (±3.1%)
(*E*)-2-undecenal	0.1 (±11.7%)	0.1 (±11.5%)	0.1 (±6.5%)	1.6 (±11.5%)	0.6 (±12.7%)	0.1 (±3.9%)	0.1 (±8.1%)	0.2 (±6.7%)	0.2 (±6.4%)	0.1 (±1.9%)
nonanoic acid	0.7 (±1.3%)	1.3 (±5.6%)	2.8 (±6.0%)	5.9 (±4.2%)	3.1 (±5.7%)	1.7 (±2.9%)	1.4 (±7.4%)	2.2 (±2.2%)	2.5 (±2.7%)	0.5 (±7.1%)
methional	0.03 (±5.6%)	0.1 (±7.1%)	0.04 (±4.7%)	0.03 (±10.4%)	0.1 (±13.1%)	0.02 (±1.5%)	0.04 (±2.4%)	0.02 (±0.6%)	0.02 (±3.1%)	0.03 (±8.1%)
acetic acid	267.9 (±2.9%)	13.7 (±4.2%)	262.0 (±4.0%)	148.4 (±6.5%)	176.9 (±4.5%)	111.3 (±5.9%)	212.4 (±2.3%)	243.9 (±3.2%)	239.2 (±1.0%)	126.6 (±3.2%)

3-methylbutanoic acid	18.6 (±5.5%)	6.4 (±5.7%)	23.3 (±6.1%)	3.1 (±4.8%)	8.2 (±3.4%)	8.9 (±1.0%)	19.8 (±2.0%)	14.4 (±7.0%)	11.1 (±6.6%)	17.0 (±3.9%)
decanoic acid	0.04 (±7.3%)	0.1 (±1.2%)	0.1 (±5.5%)	0.1 (±8.8%)	1.0 (±7.3%)	0.3 (±5.0%)	0.3 (±6.7%)	0.9 (±6.7%)	0.2 (±6.5%)	0.3 (±7.1%)
vanillin	0.5 (±12.2%)	0.4 (±13.6%)	0.6 (±3.2%)	1.8 (±11.5%)	0.7 (±11.2%)	0.4 (±7.3%)	0.7 (±6.8%)	0.4 (±12.8%)	0.5 (±10.7%)	1.3 (±3.8%)
(*E*,*E*)-3,5-octadien-2-one	0.1 (±12.4%)	0.04 (±4.2%)	0.2 (±7.9%)	1.1 (±13.8%)	0.5 (±14.6%)	0.5 (±8.1%)	0.1 (±3.1%)	0.1 (±11.6%)	0.1 (±20.1%)	0.2 (±7.5%)
hexanoic acid	21.5 (±7.9%)	7.7 (±5.3%)	36.8 (±5.4%)	734.2 (±5.8%)	111.3 (±4.7%)	55.8 (±7.6%)	26.0 (±7.3%)	72.2 (±5.4%)	42.8 (±6.3%)	6.8 (±6.4%)
octanoic acid	0.3 (±2.9%)	0.4 (±2.8%)	1.5 (±3.1%)	7.3 (±8.2%)	4.8 (±1.2%)	3.6 (±4.0%)	0.5 (±5.6%)	1.4 (±5.1%)	1.7 (±4.4%)	2.0 (±6.4%)
phenylacetic acid	0.4 (±12.4%)	nd	0.5 (±11.8%)	1.9 (±7.5%)	3.9 (±6.2%)	1.2 (±5.6%)	0.5 (±12.6%)	1.1 (±9.0%)	0.8 (±6.9%)	0.6 (±2.3%)
γ-octalactone	0.02 (±9.2%)	0.03 (±10.1%)	0.05 (±10.5%)	4.3 (±11.8%)	0.5 (±8.9%)	0.1 (±3.5%)	0.01 (±20.0%)	0.2 (±7.3%)	0.3 (±1.1%)	0.01 (±3.4%)
2-methylbutanoic acid	16.7 (±10.7%)	13.0 (±9.2%)	21.4 (±4.7%)	9.1 (±6.6%)	5.5 (±9.8%)	32.2 (±3.8%)	29.0 (±9.8%)	13.2 (±3.6%)	3.3 (±8.4%)	25.6 (±7.9%)
2,3-octanedione	0.1 (±2.9%)	0.05 (±7.6%)	0.1 (±8.9%)	0.7 (±9.3%)	0.2 (±7.1%)	0.2 (±3.1%)	0.1 (±5.3%)	0.2 (±8.1%)	0.1 (±11.4%)	0.1 (±2.1%)
2-undecanone	0.1 (±8.1%)	0.1 (±0.2%)	0.1 (±6.5%)	0.7 (±9.0%)	0.1 (±1.6%)	0.2 (±5.3%)	0.1 (±7.9%)	0.1 (±3.9%)	0.1 (±6.4%)	0.1 (±3.7%)
(*E*)-2-dodecenal	0.1 (±10.2%)	nd	nd	8.8 (±9.7%)	1.4 (±9.3%)	0.2 (±5.6%)	nd	0.2 (±11.7%)	0.3 (±10.1%)	nd
acetoin	1.0 (±1.1%)	1.0 (±1.9%)	1.0 (±0.7%)	1.1 (±1.9%)	1.0 (±1.6%)	1.0 (±1.2%)	1.0 (±0.2%)	1.0 (±0.8%)	1.0 (±0.3%)	1.0 (±2.5%)
butyric acid	nd	nd	nd	19.6 (±5.2%)	40.2 (±1.9%)	8.8 (±4.8%)	1.7 (±1.8%)	5.2 (±5.9%)	3.3 (±1.2%)	5.0 (±2.5%)
pentanoic acid	0.9 (±11.0%)	nd	2.7 (±8.4%)	59.0 (±3.9%)	34.3 (±7.1%)	5.8 (±6.9%)	nd	7.0 (±13.5%)	4.4 (±4.0%)	nd
heptanoic acid	2.2 (±3.9%)	nd	2.8 (±6.8%)	39.9 (±5.3%)	14.7 (±8.0%)	4.6 (±8.0%)	2.4 (±4.0%)	6.3 (±5.3%)	5.4 (±1.9%)	0.6 (±6.9%)
dodecanoic acid	0.03 (±9.3%)	0.3 (±10.2%)	0.3 (±12.3%)	0.4 (±4.5%)	0.8 (±4.4%)	0.3 (±6.4%)	0.3 (±4.5%)	0.4 (±9.0%)	0.3 (±5.9%)	0.03 (±6.3%)
tetradecanoic acid	1.2 (±8.6%)	5.6 (±7.0%)	2.0 (±8.6%)	4.0 (±10.1%)	7.7 (±8.6%)	3.7 (±8.8%)	2.2 (±9.6%)	3.5 (±3.1%)	3.1 (±4.0%)	1.6 (±9.0%)

Mean, Standard Deviation and Relative SD were determined based on replicate sample workup and analysis (n = 3). **A:** Nutralys F85F (Roquette, Lestrem, France) **B:** Prestige (Parrheim Foods, Saskatoon, Canada) **C:** Nutralys S85F (Roquette) **D:** Bio Erbsenprotein (Golden Peanut, Garstedt, Germany) **E:** Bio Erbsen Protein (Piowald, Mühbrook, Germany) **F:** Erbsenproteinisolat 1501018 (Döhler, Darmstadt, Germany) **G:** Pea Pro (LSP Sports Nutrition, Bonn, Germany) **H:** Empro E86HV (Emsland-Stärke, Emlichheim, Germany) **I:** Empro E86 (Emsland-Stärke) **J:** Pisane C9 (Cosucra Group, Warcoing, Belgium).

Table S5. Odor activity values (*OAV*) of the quantified odorants in different pea protein samples.

odorants	A	B	C	D	E	F	G	H	I	J
3-methylbutanal	14240	1536	10186	87052	43773	38096	18140	58160	38426	18708
hexanal	4261	1268	6202	57493	18087	14115	4827	34763	18034	5932
acetaldehyde	4980	2966	4512	8219	5029	9898	5159	7325	5825	15851
(*E,E*)-2,4-decadienal	2036	2345	3736	6779	18081	3086	2787	2599	2471	5455
phenylacetaldehyde	602	1143	1173	222	1689	2040	1368	1130	385	1850
(*E,E*)-2,4-nonadienal	943	784	1156	5354	2574	1345	966	2077	2329	865
(*E*)-2-octenal	195	67	533	4017	1185	746	244	469	530	127
diacetyl	233	91	329	550	280	552	367	301	473	704
benzaldehyde	136	101	248	952	241	979	325	398	236	175
heptanal	113	22	217	949	464	274	133	310	281	96
2-methylbutanal	261	205	178	334	973	512	296	405	294	360
(*E*)-2-undecenal	156	169	157	2091	732	127	135	195	242	98
nonanoic acid	27	49	107	226	119	65	54	86	96	19
methional	67	156	85	69	250	54	88	54	51	66
acetic acid	48	2.5	47	27	32	20	38	44	43	23
3-methylbutanoic acid	38	13	48	6	17	18	41	29	23	35
decanoic acid	13	35	15	15	281	83	72	252	59	97
vanillin	9	7	11	33	13	8	13	8	10	24
(*E,E*)-3,5-octadien-2-one	5	1.3	9	40	17	17	5	4.3	5	6
hexanoic acid	4.5	1.6	8	153	23	12	5	15	9	1.4
octanoic acid	1.7	2.0	8	38	25	19	2.7	7	9	11
phenylacetic acid	6	0	8	28	57	18	8	16	12	10
γ-octalactone	2.9	4.3	7	663	74	16	1.8	28	42	1.6
2-methylbutanoic acid	5	4.2	7	2.9	1.8	10	9	4.3	1.1	8
2,3-octanedione	2.0	1.6	4.8	24	8	7	2.6	8	4.2	2.2
2-undecanone	3.1	3.4	2.8	3.1	6	7	2.7	3.9	3.4	6
(*E*)-2-dodecenal	408	0	0	43868	6991	794	0	1193	1621	0
acetoin	1.6	1.8	1.7	1.8	1.7	1.7	1.7	1.7	1.7	1.7
butyric acid	0	0	0	8	17	3.7	0.7	2.2	1.4	2.1
pentanoic acid	0.1	0	0.2	5	3.1	0.5	0	0.6	0.4	0
heptanoic acid	0.7	0	0.9	1.3	4.9	1.5	0.8	2.1	1.8	0.2

dodecanoic acid	0	0	0	0	0	0	0	0	0	0
tetradecanoic acid	0.1	0.6	0.2	0.4	0.8	0.4	0.2	0.4	0.3	0.2

Concentrations of **Table S4** divided by the corresponding odour thresholds in water and expressed as *OAV* (Odour Activity Value). **A:** Nutralys F85F (Roquette, Lestrem, France) **B:** Prestige (Parrheim Foods, Saskatoon, Canada) **C:** Nutralys S85F (Roquette) **D:** Bio Erbsenprotein (Golden Peanut, Garstedt, Germany) **E:** Bio Erbsen Protein (Piowald, Mühbrook, Germany) **F:** Erbsenproteinisolat 1501018 (Döhler, Darmstadt, Germany) **G:** Pea Pro (LSP Sports Nutrition, Bonn, Germany) **H:** Empro E86HV (Emsland-Stärke, Emlichheim, Germany) **I:** Empro E86 (Emsland-Stärke) **J:** Pisane C9 (Cosucra Group, Warcoing, Belgium).

R script of *OAV* heatmap

```
library(readr)
library(ComplexHeatmap)

OAV_data <- read.delim("#insert OAV data")
rownames(OAV_data)<- OAV_data[,1]
OAV_data<- OAV_data[,-1]
head(OAV_data)
summary(OAV_data)
max(OAV_data)

OTs_1 <- read.delim("#insert odor thresholds")
rownames(OTs_1)<- OTs_1[,"analyte"]
OTs_1<- OTs_1[,-1]

n<- length(OTs_1$KFO)
np<- n -15
OTs_1$KFO

Bottomha_ot = HeatmapAnnotation(SensoryScore = anno_lines(log10((OTs_1$OT_ug.kg)+1), smooth
= TRUE),
                              #add_points = TRUE,
                              annotation_name_rot = c(90,0),
                              annotation_name_side = "left",
                              #height = unit(3, "cm"),
                              annotation_height = unit(c(3,0.5), "cm"),
                              annotation_name_gp= gpar(fontsize = 7),
                              KFO = anno_simple(rep(1,n) , pch = OTs_1$pch,  pt_size = unit(3, "mm"), pt_gp =
gpar(col = "blue")
                              ),
                              annotation_label = c("LOG(OT+1)", "KFO")
) #make the bar height smaller
pollo<- OTs_1$KFO
collo<- rep("white", n)

names(collo) <- OTs_1$KFO

lgd = Legend(labels = c("p<=0.05", "p=>0.05"), title = "legend", type = "points",
            pch = c(8,8), legend_gp = gpar(col = c("azure 2","azure 2")))

Bottomha_ot2 = HeatmapAnnotation(SensoryScore = anno_lines(log10((OTs_1$OT_ug.kg)+1),
smooth = TRUE),
                               annotation_name_rot = c(90,0),
                               annotation_name_side = "left",
                               annotation_height = unit(c(3,0.5), "cm"),
                               annotation_name_gp= gpar(fontsize = 7),
                               KFO = anno_simple(OTs_1$KFO,
                                              pch = OTs_1$pch,
                                              col = collo
                               ),
                               # pt_size = unit(3, "mm"),
                               #pt_gp = gpar(col = "blue"),
                               annotation_label = c("LOG(OT+1)", "KFO")
)

top_pp = HeatmapAnnotation(AromaClass = (OTs_1$class),
                         col = list(
                           AromaClass = c("aldehyde" = "#9e9ac8", "ketone" = "#fdb863", "acid" = "#1a9641",
"lactone" = "#a6d96a")
                         ),
```

```
                        show_annotation_name = FALSE,
                        border = TRUE
)
cc<-log(OAV_data+1)

HM<- Heatmap(as.matrix(t(log(OAV_data+1))),
            name = "OAV ",
            #column_title = "bbbbbbbbbbbbbbbbb",
            #border = TRUE,
            #column_title_gp = gpar(fill = "#b2df8a", border = "#b2df8a"),
            col = colorRamp2(c(0, 1, 2, 4, 5, 6, 8, 10, 12),
                            c("white",
                              "#ffffe5",
                              "#fff7bc",
                              "#fee391",
                              "#fec44f",
                              "#fe9929",
                              "#ec7014",
                              "#cc4c02",
                              "#8c2d04")),
            heatmap_legend_param = list(title = "Log OAV",
                                        at = c(0, 1, 2, 4, 6, 8, 10, 12, 15)
            ),
            row_title = "Pea protein isolates",
            column_title = "Odour active compounds",
            cluster_rows = TRUE,
            cluster_columns = TRUE,
            row_split = 2,
            #column_split = 2,
            row_dend_width = unit(2, "cm"),
            top_annotation = top_pp,
            bottom_annotation = Bottomha_ot2)

HM
```
